# Prioritizing single-nucleotide variations that potentially regulate alternative splicing

**DOI:** 10.1186/1753-6561-5-S9-S40

**Published:** 2011-11-29

**Authors:** Mingxiang Teng, Yadong Wang, Guohua Wang, Jeesun Jung, Howard J Edenberg, Jeremy R Sanford, Yunlong Liu

**Affiliations:** 1 School of Computer Science and Technology, Harbin Institute of Technology, Harbin, Heilongjiang, China; 2 Department of Medical and Molecular Genetics, Indiana University School of Medicine, 410 West 10th Street, Suite 5000, Indianapolis, IN 46202, USA; 3 Center for Computational Biology and Bioinformatics, Indiana University School of Medicine, 410 West 10th Street, Suite 5000, Indianapolis, IN 46202, USA; 4 Department of Biochemistry and Molecular Biology, Indiana University School of Medicine, 635 Barnhill Drive, MS 4053, Indianapolis, IN 46202, USA; 5 Center for Medical Genomics, Indiana University School of Medicine, 1345 West 16th Street, Indianapolis, IN 46202, USA; 6 Department of Molecular, Cellular, and Developmental Biology, University of California, 1101 Pacific Avenue, Suite 200, Santa Cruz, CA 95060, USA

## Abstract

Recent evidence suggests that many complex diseases are caused by genetic variations that play regulatory roles in controlling gene expression. Most genetic studies focus on nonsynonymous variations that can alter the amino acid composition of a protein and are therefore believed to have the highest impact on phenotype. Synonymous variations, however, can also play important roles in disease pathogenesis by regulating pre-mRNA processing and translational control. In this study, we systematically survey the effects of single-nucleotide variations (SNVs) on binding affinity of RNA-binding proteins (RBPs). Among the 10,113 synonymous SNVs identified in 697 individuals in the 1,000 Genomes Project and distributed by Genetic Analysis Workshop 17 (GAW17), we identified 182 variations located in alternatively spliced exons that can significantly change the binding affinity of nine RBPs whose binding preferences on 7-mer RNA sequences were previously reported. We found that the minor allele frequencies of these variations are similar to those of nonsynonymous SNVs, suggesting that they are in fact functional. We propose a workflow to identify phenotype-associated regulatory SNVs that might affect alternative splicing from exome-sequencing-derived genetic variations. Based on the affecting SNVs on the quantitative traits simulated in GAW17, we further identified two and four functional SNVs that are predicted to be involved in alternative splicing regulation in traits Q1 and Q2, respectively.

## Background

Alternative splicing is an important level of gene regulation and greatly contributes to proteome diversity [1]. In humans, more than 90% of genes encode multiple protein isoforms [2], and many diseases are caused by the disregulation of splicing patterns [3,4]. In eukaryotic cells, splicing patterns are tightly regulated in a temporospatial manner through a set of RNA-binding proteins (RBPs) that bind to *cis*-acting sites on the precursor mRNA (pre-mRNA). With the advent of microarray and next-generation sequencing technology, the RNA-binding consensus sequences of several RBPs have recently been identified [5-8].

Nonsynonymous single-nucleotide polymorphisms (SNPs) alter the amino acid composition of a protein; their effects on protein functions can be predicted by many bioinformatics tools, including SIFT [9], PolyPhen [10], SNPs3D [11], and MAPP [12]. Nonsynonymous SNPs contribute to the etiology of many diseases [13]. Recent studies, however, suggest that synonymous SNPs in exons are also functionally important [14]. These variations frequently affect the binding of splicing regulatory factors (SRFs) and potentially result in abnormal pre-mRNA splicing patterns. We have previously reported a transcriptome-wide profiling of SFRS1 protein, a highly conserved, essential pre-mRNA splicing factor with dual functions in constitutive and alternative splicing [7]. A search for the SFRS1 consensus motif within the Human Gene Mutation Database identified 181 mutations in 82 different genes that disrupt the predicted SFRS1 binding sites [7].

In this study, we present a computational strategy to systematically characterize the potential capability of single-nucleotide variations (SNVs) to regulate alternative splicing. Focusing on the exonic SNVs identified in each of the seven populations in the 1000 Genomes Project, we found that the minor allele frequencies (MAFs) for the synonymous SNVs residing in alternatively spliced exons and potentially disrupting the binding of RBPs are more comparable to nonsynonymous SNVs than to other SNVs, suggesting that they are under similar selection pressure. This result suggests the importance of regulatory SNVs that are associated with certain diseases or phenotypes and the importance of integrating biological annotation into genetic association studies. Furthermore, from the SNVs that are simulated to be associated with the phenotypes distributed by Genetic Analysis Workshop 17 (GAW17) [15], we identified two and four regulatory candidates for phenotypes Q1 and Q2, respectively.

## Methods

### Data

Genotypes of SNVs for 697 individuals from seven populations (CEPH [European-descent population from Utah], Denver Chinese, Han Chinese, Japanese, Luhya, Tuscan, and Yoruba) were derived from the sequence alignment files created in the 1000 Genomes Project and distributed by GAW17. We considered 24,487 exonic SNVs (both synonymous and nonsynonymous) within 3,205 autosomal genes. For phenotypes, the GAW17 committee carried out 200 replicate simulations on traits Q1 and Q2 and disease liability for 697 individuals; the simulations were based on an answer sheet of associated SNVs for each trait [15].

### Assessing the capability of a genetic variation to change the binding affinity of an RNA-binding protein

As a proof of concept, we focus our analysis on nine RBPs whose binding affinity has been characterized using an in vitro assay called RNAcompete [8]. Using a customized microarray that contains all the potential 7-base and 8-base sequences and a single binding reaction, this technology determines the relative preferences of RBPs for short RNA sequences. For each of the nine RBPs being studied (HuR, Vts1, FUSIP1, PTB, U1A, SF2/ASF, SLM2, RBM4, and YB1), a preference score is provided for every possible RNA 7-mer, indicating the level of binding affinity of the specific RBP-7mer RNA pair [8].

**Figure 1:**
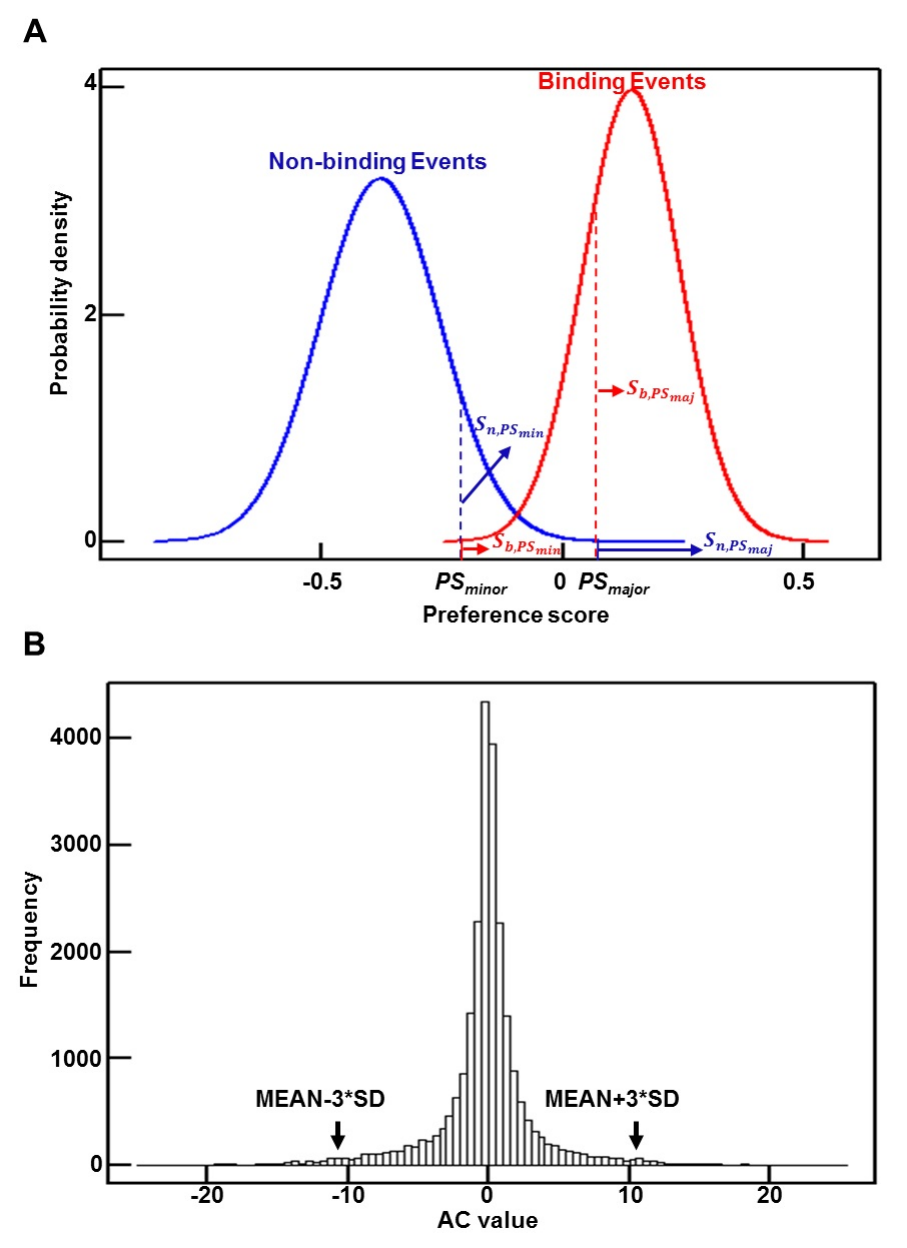
**Computational strategy** (A) Assessing the capability of a SNV to change the binding affinity of an RBP. (B) Distribution of AC values for SF2.

To evaluate the potential of a SNV to alter the binding affinity of an RBP, for each RBP we first characterized the distribution of the preference scores for the binding and nonbinding events; a higher preference score indicates higher binding potential. We used the mean plus 3 times the standard deviation as the cutoff to distinguish binding and nonbinding events. As indicated in Figure 1A, the preference score distributions for binding and nonbinding events form two normal-like distributions. For each SNV, we retrieved the highest preference score for the seven possible 7-mers that contain the variation and calculated the binding preference scores for the major allele and minor allele separately. We then used the following strategy to calculate the capability of one SNV to change the binding affinity of an RBP:(1)

where *PS*_min_ and *PS*_maj_ are the RBP’s preference score for the minor allele and the major allele, respectively; *b* and *n* denote score distributions for the binding and nonbinding events, respectively; and  is the area to the right of the preference score on the minor allele (*PS*_min_) under the distribution indicating binding events (*b*). A positive or negative AC value indicates that the minor allele will cause a gain or loss of binding affinity, respectively.

### Alternative splicing events

We used the AltEvent track in the UCSC (University of California, Santa Cruz) Genome Browser to identify the SNVs residing in the exons that can be alternatively spliced [16]. This track documents various types of alternative splicing that result in more than one gene isoform. We consider here the SNVs labeled to be associated with alternatively spliced exons by the UCSC Table Browser. These variations are more likely to be associated with the splicing regulation.

### GAW17 data analysis

We calculated the capability of changing the binding affinity of each RBP for all 24,487 exonic SNVs, based on the AC score (Eq. (1)), which is defined as the logarithmic ratio of the binding likelihood of the minor and major alleles. A more extreme positive or negative AC value indicates a higher possibility of altering an RBP’s binding affinity (gain or loss of the binding). For each RBP, the AC values for all the exonic SNVs follow a normal-like distribution; one example, for SFRS1 (or SF2), is shown in Figure 1B. We consider the outliers, the SNVs with AC values larger than the mean plus 3 times the standard deviation or less than the mean minus 3 times the standard deviation, as candidates that may change the binding affinity of the RBP.

## Results and discussion

### Many synonymous variations potentially change the binding of RBPs

**Figure 2:**
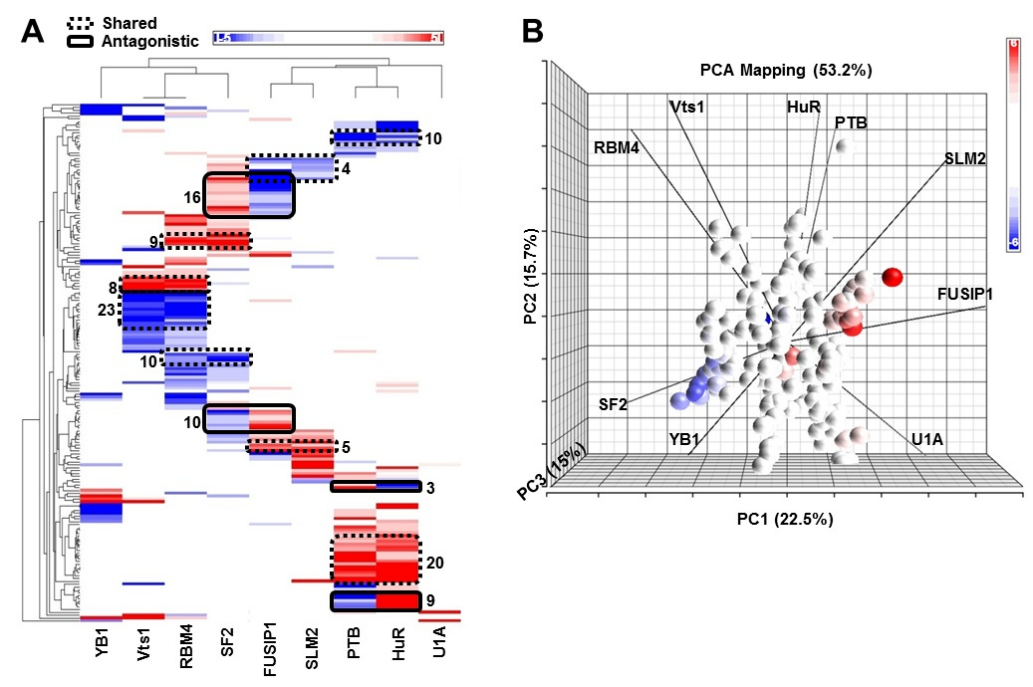
**Relationships between synonymous variations and their effects on the binding of RBPs** (A) Hierarchical clustering of RBPs and 183 synonymous variations that can potentially affect binding of more than one RBP, based on their AC values (Eq. (1)); red and blue indicate potential gain and loss of binding in the minor alleles. (B) Principal components analysis (PCA) biplot with variations plotted against the top three principal components; nine RBPs show their similarity or dissimilarity with respect to the effects of variations on their binding affinity.

To illustrate the regulatory role of SNVs, we focused our analysis on 10,113 synonymous SNVs, of which 1,851 candidates potentially affect the binding of at least one of the nine RBPs. Interestingly, some of the variations potentially affect the binding of more than one RBP: 173 variations affect bindings of 2 RBPs and 10 variations affect 3 RBPs. We clustered these 183 synonymous SNVs based on their AC values. Clearly, some of the SNVs can potentially cause the gain or loss of the binding of two RBPs. For instance, Vts1 and RBM4 share 32 SNVs in the heat map (Figure 2A). Similar relationships can be found between RBM4 and SF2, between FUSIP1 and SLM2, and between PTB and HuR. Interestingly, some SNVs (e.g., the SF2-FUSIP1 pair) can cause antagonistic effects on the binding of two RBPs: the loss of the binding of one RBP and the gain of another one. For the PTB-HuR pair, some variations can cause the loss or gain of binding to both RBPs, whereas others can cause the gain of PTB with the loss of HuR. The relationships between binding changes to the nine RBPs are shown in the principal components analysis (PCA) biplot [17] (Figure 2B). In this figure each dot represents one of the 183 synonymous SNVs in the first three principal components, and the nine lines represent the nine RBPs, using their weights for the components. Biplots provide one way to use the correspondence between the SNV-like and RBP-like patterns revealed by PCA to identify groups of RBPs that have binding that is characteristic of a group of variations. The angles between two RBPs indicate the similarity of the effect of variations on their binding affinity. For instance, both the Vts1-RBM4 and the HuR-PTB pairs show similar directionality, whereas the line for SF2 is almost opposite to the direction of FUSIP1.

### Synonymous variations that potentially regulate alternative splicing show lower minor allele frequency

**Figure 3:**
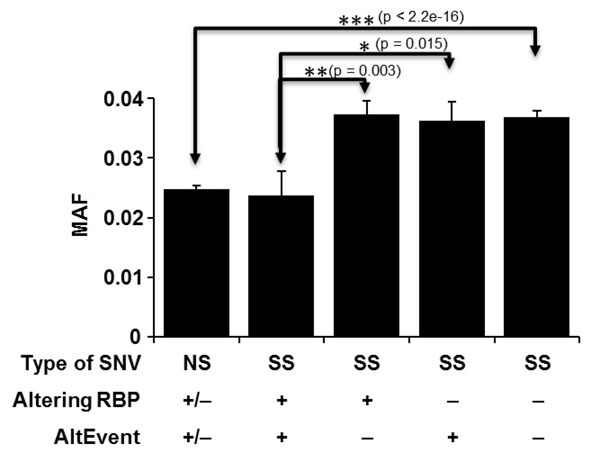
Comparisons of MAFs among different categories of SNVs according to 697 individuals

To characterize the disease relevance of predicted synonymous SNVs that potentially affect RBP binding, we further divided all the synonymous SNVs into four (nonoverlapping) categories: (1) synonymous SNVs affecting RBP binding but not located within alternatively spliced exons (SS+RBP−Alt), (2) synonymous SNVs located within alternatively spliced exons but not affecting RBP binding (SS+Alt−RBP), (3) synonymous SNVs not only affecting RBP binding but also located within alternatively spliced exons (SS+RBP+Alt), and (4) synonymous SNVs located outside alternatively spliced exons and not affecting any RBP binding (SS−RBP−Alt). We examined the MAFs of the SNVs in the different categories based on all 697 individuals distributed by GAW17. We found that the average MAFs for the category SS+RBP+Alt are similar to the ones for the nonsynonymous SNVs in all the populations and that the average MAFs for the other three categories are markedly higher (Figure 3). Student’s *t* test was used to quantitatively measure the significance of MAF differences between different categories; *p*-values are labeled in Figure 3. In addition, the MAFs show that the trends in each of the seven individual populations are similar to those in all the individuals (data not shown). This result indicates that the synonymous SNVs within alternatively spliced exons that affect RBP binding are under similar selection as the nonsynonymous SNVs and may contribute to the disease by influencing the splicing patterns. However, it should be noted that experimental validation is required to truly identify the functions of these SNV candidates (SS+RBP+Alt). We have also examined the MAFs of the nonsynonymous variations that potentially affect alternative splicing. We have not seen significant differences compared to other nonsynonymous variations (data not shown). This suggests that amino-acid-changing SNVs are under similar selection pressure regardless of their splicing regulating potentials.

### Prioritizing phenotype-associated variations that regulate alternative splicing

**Figure 4:**
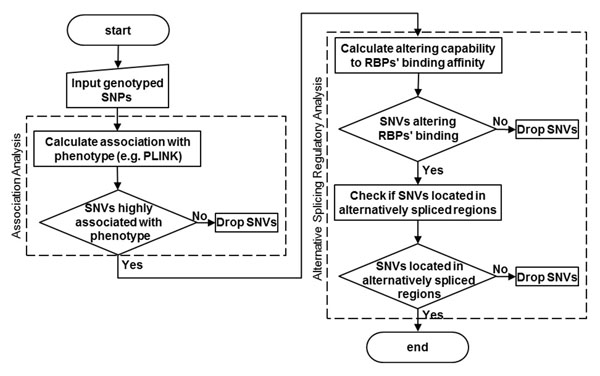
Proposed workflow

Based on our observations, we propose a workflow to prioritize variations identified in the exome sequencing (Figure 4). First, those SNVs associated with the phenotype can be selected on the basis of a statistical procedure. Then, to prioritize these, we first predict whether the identified single-nucleotide variation will affect the binding of RBPs using the strategy presented in the Methods section. Among the SNVs that affect RBP binding, we select those located within a known alternatively spliced exon, using the AltEvent track in the UCSC Genome Browser or other bioinformatics tool. The variations that meet all criteria are candidates for functional SNVs that potentially affect the phenotype.

Distributed by GAW17, each of traits Q1, Q2, and disease liability are simulated to be affected by a particular set of nonsynonymous SNVs [15]. We further checked whether these SNVs can also potentially regulate alternative splicing by affecting the binding of RNA-binding proteins, using the outlined workflow. We found that two and four SNVs were involved in alternative splicing in traits Q1 and Q2, respectively (Table 1); no SNVs were found for disease liability. This result suggests that the nonsynonymous SNVs may affect the traits both by causing amino acid substitutions and by regulating splicing patterns. Without loss of generalizability, synonymous regulatory SNVs for phenotypes can also be prioritized if phenotype association is carried out first to obtain a set of associated candidates.

**Table 1:** Alternative splicing regulatory SNVs in Q1 and Q2

SNV	Gene	RBP	Minor allele function	Trait
C1S6537	*ARNT*	Vts1	Gain binding	Q1
C1S6540	*ARNT*	RBM4	Loss binding	Q1
C3S4860	*BCHE*	HuR	Loss binding	Q2
C3S4874	*BCHE*	SLM2	Gain binding	Q2
C8S1799	*PLAT*	RBM4/Vts1	Loss/loss binding	Q2
C6S5449	*VNN3*	RBM4	Gain binding	Q2

## Conclusions

We present a strategy to prioritize synonymous SNVs based on their likely capacity to change the binding affinity of an RBP and thereby affect pre-RNA splicing. Synonymous variations within alternatively spliced exons that affect RBP binding appear to be under similar selection pressure as nonsynonymous SNVs and therefore are candidates for functional SNVs affecting the phenotype. Synonymous SNVs that are outside the AltEvent or that do not affect RBP binding are under less selection pressure and therefore are considered less likely to be functional. We also show that some RBPs may have antagonistic relations when binding to synonymous variations, whereas others share the common consequence of gain or loss of binding. Trait-specific regulatory SNVs indicate that some nonsynonymous SNVs not only result in amino acid substitution but also regulate alternative splicing.

Our proposed workflow provides an applicable way to identify phenotype-associated variations involved in alternative splicing. It should be noted that both nonsynonymous and synonymous variations that affect alternative splicing can be identified by using this workflow. The results of this analysis will lead to novel hypotheses for investigating the mechanisms of disease-causing mutations.

The nine RBPs analyzed in this study are properly assessed for their binding preferences on RNA 7-mers [8]. Without losing generalizability, the same strategy can be applied to other RBPs or to microRNA binding sites. With the technological advent of next-generation sequencing and the application of CLIP (crosslinking immunoprecipitation)-seq or RIP-seq technologies [7,18,19], binding affinities of more RBPs will be available. This will provide greater opportunity to understand the genetic mechanisms of disease.

## Competing interests

The authors declare that there are no competing interests.

## Authors’ contributions

MT, YW and YL contributed to the design of the study. MT, YW and YL designed and performed the computational modelling and drafted the manuscript. GW, JJ, HJE, JRS, and YW participated in coordination, discussions related to result interpretation and revision of the manuscript. All authors read and approved the final manuscript.
